# Supplementary figures and images for: Pharmacological inhibition of mTORC1 reduces neural death and damage volume after MCAO by modulating microglial reactivity
Source: Biol Direct. 2024 Apr 6;19:26. doi: 10.1186/s13062-024-00470-5 (PMC10999095; doi:10.1186/s13062-024-00470-5)

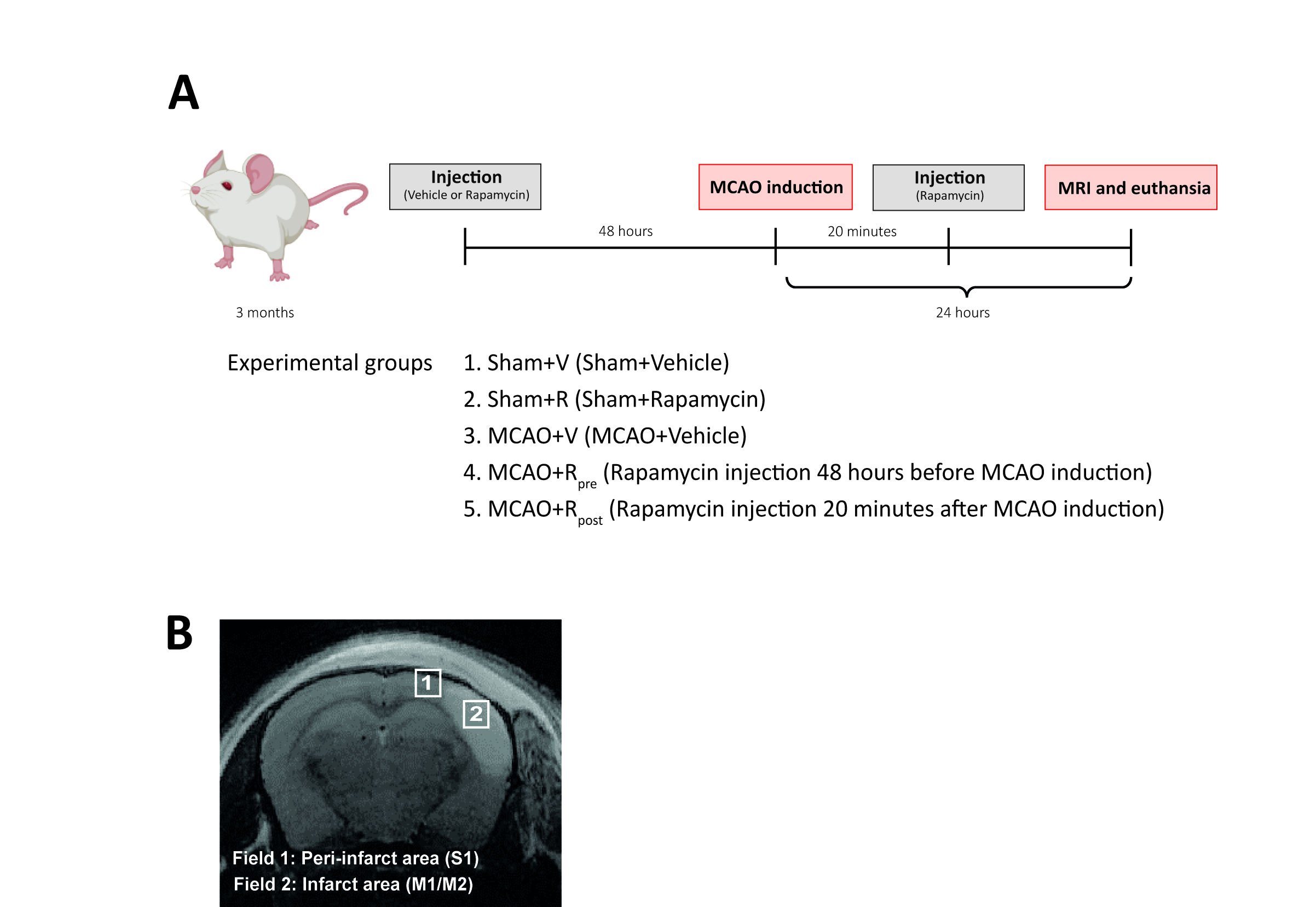

Supplement: Supplementary file 1 — Additional file 1. Fig. S1. Experimental design and determination of damage regions. A) Mice were randomly distributed into five experimental groups. Sham groups were injected with the vehicle solution or rapamycin 48 hours before MCAO simulation. MCAO+V group was injected with the vehicle solution 48 hours before the surgery. MCAO+Rpre group was injected with rapamycin 48 hours before the surgical procedure, while the MCAO+Rpost group was injected with rapamycin 20 minutes after the surgery. Twenty-four hours following the MCAO simulation or induction, brain damage was assessed by MRI. Then animals were euthanized collecting brains to perform the corresponding analysis. B) According to the MRI images, infarcted area affects to M1/M2 cortex (Field 2) whereas the peri-infarct area corresponds to the S1 cortex (Field 1). [file 13062_2024_470_MOESM1_ESM.tif]

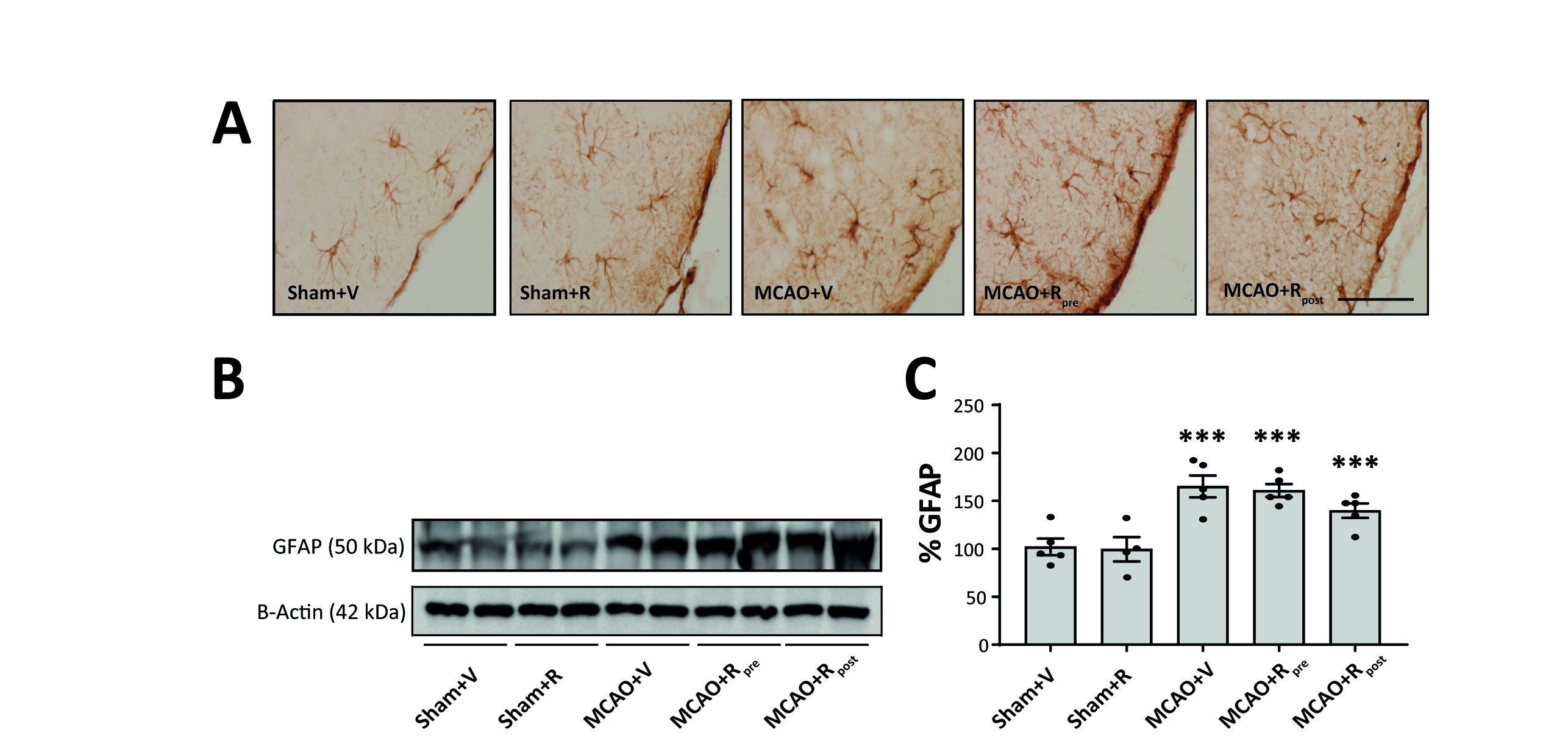

Supplement: Supplementary file 2 — Additional file 2. Fig. S2. MCAO triggers astrocytic response. A) Representative immunohistochemical images from the entire damage area (coronal section 10 µm). Staining was performed used GFAP antibody. Scale bar: 25 µm. B) Representative Western Blot images of the analysed extracts proteins from mice cerebral cortex. Immunodetections were performed using antibodies against GFAP and B-Actin, as a loading control. C) Graphical quantification of immunodetection by Western Blot. Data are normalized against B-Actin and expressed as the percentage of variation versus sham. Graph values represent means ± SEM (Ordinary one-way ANOVA *p ≤ 0.05; **p ≤ 0.01; ***p ≤ 0.001). Sham+V n=5, Sham+R n=4, MCAO+V n=5; MCAO+Rpre n=5 and MCAO+Rpost n=5 (V=vehicle; R=rapamycin). * vs Sham+V. [file 13062_2024_470_MOESM2_ESM.tif]
